# Monoglyceride lipase gene knockout in mice leads to increased incidence of lung adenocarcinoma

**DOI:** 10.1038/s41419-017-0188-z

**Published:** 2018-01-18

**Authors:** Renyan Liu, Xin Wang, Christopher Curtiss, Steve Landas, Rong Rong, M. Saeed Sheikh, Ying Huang

**Affiliations:** 1 Department of Pharmacology, Upstate Medical University, State University of New York, Syracuse, NY 13210 USA; 2 Department of Pathology, Upstate Medical University, State University of New York, Syracuse, NY 13210 USA; 3 Department of Pathology and Anatomical Sciences, University at Buffalo, State University of New York, Buffalo, NY 14214 USA

## Abstract

Monoglyceride lipase (MGL) is a recently discovered cancer-related protein. The role of MGL in tumorigenesis remains to be fully elucidated. We have previously shown that MGL expression was reduced or absent in multiple human malignancies, and overexpression of MGL inhibited cancer cell growth. Here, we have generated the MGL knockout mice to further investigate the role of MGL in tumorigenesis in vivo. Our results indicate that MGL-deficient (MGL^+/−^, MGL^−/−^) mice exhibited a higher incidence of neoplasia in multiple organs, including the lung, spleen, liver and lymphoid tissues. Interestingly, lung neoplasms were the most common neoplastic changes in the MGL-deficient mice. Importantly, MGL-deficient animals developed premalignant high-grade dysplasia and adenocarcinomas in their lungs. Investigation of the MGL expression status in lung cancer specimens from patients also revealed that MGL expression was significantly reduced in the majority of primary human lung cancers when compared to corresponding matched normal tissues. Furthermore, mouse embryonic fibroblasts (MEFs) from MGL-deficient animals showed characteristics of cellular transformation including increased cell proliferation, foci formation and anchorage-independent growth. Our results also indicate that MGL deficiency was associated with activation of EGFR and ERK. In addition, pro-inflammatory molecules COX-2 and TNF-α were also activated in the MGL-deficient lung tissues. Thus, our results provide new insights into the novel role of MGL as an important negative regulator of EGFR, COX-2 and TNF-α. Accordingly, EGFR and COX-2/TNF-α activation/induction is expected to play important roles in MGL deficiency-driven lung tumors. Collectively, our results implicate the tumor suppressive role of MGL in preventing tumor development in vivo, particularly in context to the lung cancer, and highlight its role as a potential tumor suppressor.

## Introduction

Lung cancer is the leading cause of cancer mortality for both men and women worldwide^1^. From both histologic and therapeutic perspectives, lung carcinomas are traditionally divided into small-cell lung carcinoma (SCLC) and non-small-cell lung carcinoma (NSCLC). NSCLC represents the majority (>85%) of lung carcinomas that is further subclassified as adenocarcinoma (50%), squamous cell carcinoma (~40%) and large cell carcinoma (~10%)^2^. Squamous cell carcinoma often arises in the proximal airways and is largely associated with smoking. Adenocarcinoma often arises in the peripheral lung, and while adenocarcinoma does occur in smokers, an increasing proportion of adenocarcinomas are diagnosed in non-smokers and women^2,3^.

Despite advances in chemotherapy, NSCLC remains difficult to cure because of poor understanding of the pathological mechanisms underlying this disease. Studies have demonstrated that epidermal growth factor receptor (EGFR) is important in lung cancer development. EGFR gene mutation, amplification and overexpression have been found in a significant portion of NSCLCs^2^. Increased EGFR activity is associated with frequent lymph node metastases, insensitivity to chemotherapy and poor survival^4,5^. Activating mutations or overexpression of EGFR leads to activation of downstream oncogenic signals, i.e., the extracellular signal-regulated kinase (ERK) and phosphatidylinositol-3-kinase/Akt pathways. Thus, EGFR has become an important therapeutic target for lung cancer treatment. The molecular mechanisms responsible for NSCLC remain to be fully elucidated. Accordingly, a better understanding of the molecular alterations underlying this disease is critical for accurate pathological diagnosis, and development of effective targeted therapies to improve patient survival.

Our previous study had suggested monoglyceride lipase (MGL) to be a potential tumor suppressor that could play an important role in tumorigenesis^6^. We had shown that MGL messenger RNA (mRNA) expression was reduced or absent in multiple human malignancies. For example, 16 out of 21 (76%) lung cancer patients showed reduced or absent MGL mRNA levels in their tumor tissues compared to matching normal tissues^6^. MGL mRNA reduction was also noted in cancers of the colon (59.4%), rectum (50%), stomach (50%), breast (61%) and ovary (50%)^6^. Expression of exogenous MGL in colon (HCT116) and lung (H1299) cancer cells, having inherently undetectable or low MGL levels, suppressed their growth^6^. Thus, in vitro findings of our previous study^6^ indicated that MGL appeared to exhibit important growth regulatory functions of a potential tumor suppressor.

We undertook this study to further investigate the role of MGL in tumorigenesis in animals and to that end generated MGL knockout mice. Our results show that MGL-deficient (MGL^+/−^, MGL^−/−^) mice exhibit a significantly higher incidence of neoplasia in multiple organs, especially in lung, spleen and liver. Our mechanistic studies demonstrate that MGL depletion activates several important oncogenic signals. Importantly, MGL appears to act as a suppressor of lung tumorigenesis that could also serve as a target for developing newer therapeutics to manage this malignancy.

## Results

### MGL-deficient mice exhibit higher tumor incidence in multiple organs

Using the germline MGL gene targeting approach, we generated the MGL-deficient mice lacking one MGL allele (MGL^+/−^) or both (MGL^−/−^) in all tissues. The strategy used for MGL gene targeting is outlined in Supplementary Figure 1A and Materials and methods. Mice of all MGL genotypes used in our study have a mix genetic background (Supplementary Figure 1A). Genotypes of all mice used in our studies were confirmed via DNA-PCR and/or protein analyses performed on tail, lung or embryonic fibroblasts. The representative results of the genomic DNA-PCR, shown in Supplementary Figure 1B, highlight the expected PCR products from the DNAs corresponding to the MGL^+/+^, MGL^+/−^ and MGL^−/−^ genotypes. Supplementary Figure 1C shows that MGL protein levels were absent in tissues from MGL^−/−^ mice and lower in the tissues from MGL^+/−^ mice compared to those in the wild-type animals. MGL protein expression was noted in all tissues of the wild-type mice (MGL^+/+^) (Supplementary Figure 1D).

**Fig. 1:**
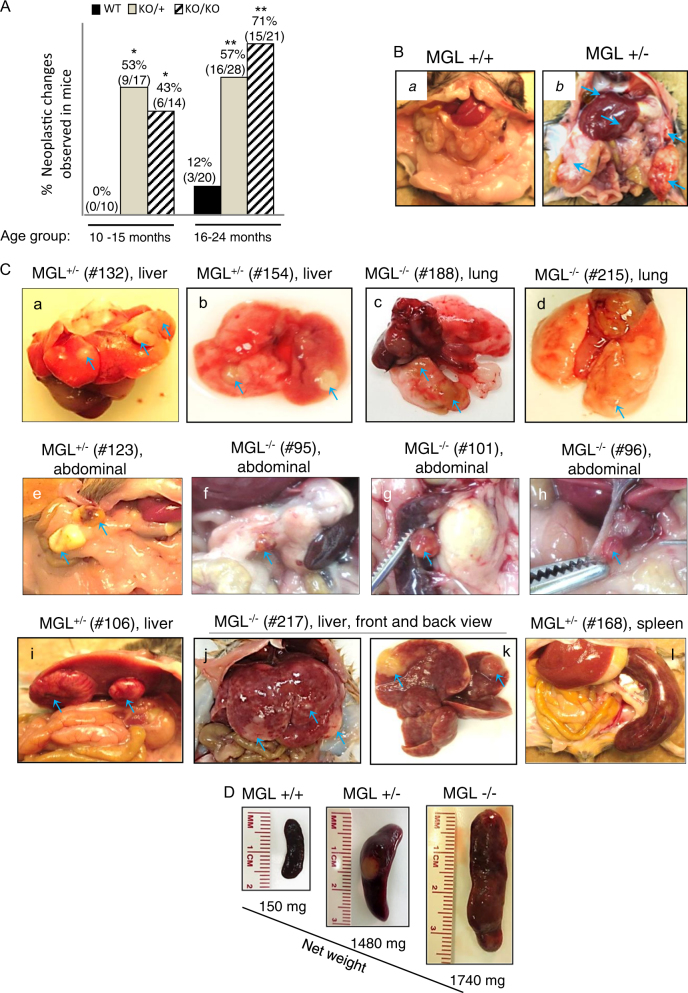
MGL-deficient mice exhibit higher tumor incidence in multiple organs. **a** Quantification of grossly visible tumor nodules observed in all organs of mice with different MGL genotypes. Each number represents an individual mouse examined. *P-value <0.05, **P-value <0.001. The statistical powers are 0.743 for 10–15-month group and 0.972 for 16–24-month group. **b** Left panel shows an image of normal viscera of MGL^+/+^ mouse without visible tumor. Right panel displays a MGL^+/−^ mouse with multiple tumor nodules (indicated by arrows) in liver, spleen and abdominal tissues. **c** Photographic images of grossly observed tumors in various organs in MGL-deficient animals; splenomegaly is also shown. The numbers of individual animals are as indicated. Blue  arrows indicate the tumor nodules. **d** Photographic images of normal-sized and enlarged spleens from MGL wild-type and MGL-deficient animals. The weights of corresponding spleens are also indicated

MGL^+/−^ and MGL^−/−^ mice were viable, fertile and did not show significant differences in terms of overall appearance and movement compared to their wild-type littermates. Crossing MGL^+/−^ mice produced MGL^+/−^, MGL^−/−^ and MGL^+/+^ pups with expected Mendelian ratios. Initially, we did not observe visible tumors in animals under 6 months of age; accordingly, in the later experiments, older animals (>10 months) were analyzed. We defined a visible tumor-like nodule as a solid (not fluid-filled) mass, white or brown in color, protruding from the surrounding tissue. Tissues (lung, liver, kidney, intestine, spleen, skin, muscle and brain) from euthanized animals of all MGL genotypes at the comparable age were assessed blindly for tumor formation microscopically by at least two pathologists, regardless of whether tumor nodules were observed. As shown in Fig. 1a, between the ages of 10–15 months, none (0/10) of the MGL^+/+^ mice had any grossly visible tumor, whereas 53% (9/17) (*p* < 0.05) of MGL^+/−^ mice and 43% (6/14) (*p* < 0.05) of MGL^−/−^ mice had visible tumor nodules in several different organs (Fig. 1a and Table 1). In the older mice (15 to 24 months), visible tumor nodules were seen in 57% (16/28) (*p* < 0.001) and 71% (15/21) (*p* < 0.001) of MGL^+/−^ and MGL^−/−^ mice, respectively, whereas only 3 out 20 (12%) wild-type (MGL^+/+^) animals had visible tumor nodules (Fig. 1a). These results indicate that MGL deficiency leads to significantly higher incidence of tumor formation in animals.

**Table 1:** Nodules and splenomegaly observed grossly in all mice examined

MGL status	No. of mice examined	No. and % mice affected	Organs and observed changes	No. of mice with indicated changes^a^
+/+	*N* = 30	3 (10%)	Lung nodules	1
			Splenomegaly	1
			Liver and spleen nodules	1
KO/+	*N* = 45	25 (56%)*	Lung nodules	11
			Splenomegaly, some with nodules	10
			Liver nodules	4
			Abdominal nodules	4
			Neck nodules	3
			Perianal mass	1
			Subcutaneous mass	1
KO/KO	*N* = 35	21 (60%)*	Lung nodules	9
			Splenomegaly, some with nodules	10
			Liver nodules	3
			Abdominal nodules	4
			Cervical/vaginal mass	1
			Oral mass	1

^a^ Some animals exhibited tumor nodules in multiple organs. *P<0.001

**Fig. 2:**
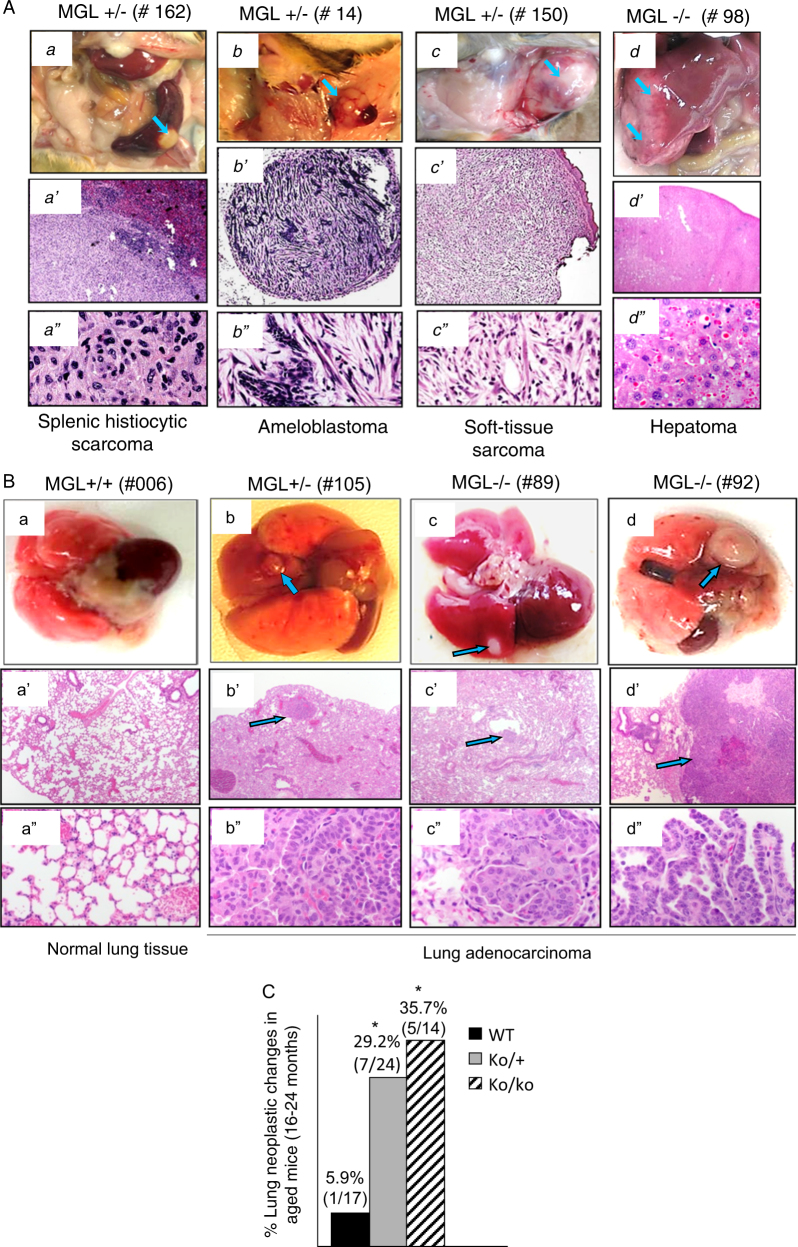
Histological examinations of tumors detected in the lungs and other tissues of MGL-deficient animals. **a** Upper panels (a, b, c, d) show photographs of the tumor nodules in various organs of several different MGL-deficient animals. Individual animal number is as indicated. Middle (4×) and lower (40×) panels show photomicrographs of hematoxylin and eosin (H&E)-stained tissue sections of corresponding tumors. **b**, **c** MGL-deficient mice show high incidence of lung adenocarcinoma compared to their wild-type littermates. **b** Lung tissues from a wild-type MGL mouse (a) and three MGL-deficient mice (b, c, d) are shown. Blue arrows indicate visible tumors (top panel, b, c, d) and their corresponding microscopic images in the middle panel (b’, c’, d’ (4×)). Bottom panel shows microscopic images at a higher magnification (40×). Images in a, a’, a” are from a wild-type MGL mouse. **c** Comparisons of mice with neoplastic changes in the lungs as noted in the wild-type MGL or MGL-deficient mice of the 16–24-month age group. Each number represents an individual mouse. *P-value <0.05. The statistical power is 0.515

In addition to increased incidence of tumorigenesis, the MGL^+/−^ and MGL^−/−^ animals developed tumors in multiple organs (Table 1). Figure 1b, left panel, shows a normal (MGL^+/+^) mouse without any visible tumor and the right panel highlights an MGL^+/−^ mouse with multiple tumor nodules in the liver, spleen and abdominal tissues. Figure 1c shows representative photographs of tumor nodules derived from lung, abdomen, liver and spleen of the MGL-deficient mice. Pathological examinations were also subsequently performed (Fig. 2). In general, normal MGL^+/+^ mice had spleens that measured 1.2–1.7 cm in length. On the other hand, MGL-deficient mice displayed significantly higher incidence of splenomegaly with spleens measuring 2–4 cm. For example, 10/45 (22%) MGL^+/−^ and 10/35 (29%) MGL^−/−^ mice displayed splenomegaly, whereas only 1/30 (3%) wild-type (MGL^+/+^) mice had splenomegaly (Table 1 and Fig. 1d).

Initial evaluation of the animals exhibiting splenomegaly revealed that the common abnormality in these animals was follicular lymphoid hyperplasia. Although some animals with splenomegaly also had only hyperproliferative changes with no tumors, splenic sarcoma and lymphoma were also noted in some animals. The detailed pathological evaluations of the animals exhibiting splenomegaly are currently ongoing and the outcome would provide more information about the role of MGL in hematological changes.

Figure 2a shows the photographs of the tumors derived from different organs of several MGL-deficient mice with gross appearance (top panels) and corresponding histology (lower panels). Histopathological examinations confirmed these tumors to be splenic sarcoma (a-a”), ameloblastoma (b-b”), soft-tissue sarcoma (c-c”) and hepatoma (d-d”).

### Lung neoplasia is the predominant abnormality in MGL-deficient mice

Interestingly, we found that MGL^+/−^ and MGL^−/−^ mice exhibited a significantly higher incidence of neoplastic changes in the lungs when compared to their wild-type littermates. For example, only 1/30 (3%) MGL^+/+^ mice exhibited lung tumor nodules, whereas 11/45 (24%) MGL^+/−^ and 9/35 (26%) MGL^−/−^ animals had lung tumor nodules (Table 1). Lung tissue sections from all euthanized animals with or without visible nodules were evaluated by two pathologists. Analyses of animals in the 10–24-month age group revealed that only 1 out of 26 (3.8%) wild-type (MGL^+/+^) animals had adenocarcinoma and none had dysplastic epithelial changes. By contrast, 7 out of 39 (17.9%) MGL^+/−^ animals (*p* < 0.05) and 5 out of 31 (16.1%) MGL^−/−^ animals (*p* < 0.07) in the same age group exhibited adenocarcinoma (Fig. 2b) or high-grade glandular dysplasia in the bronchiolar epithelium (data not shown). In the 16–24-month age group, the numbers of MGL-deficient animals with lung neoplasia were significantly higher. For example, 7 out of 24 (29.2%) MGL^+/−^ animals developed lung neoplasia (2 high-grade glandular dysplasia and 5 adenocarcinoma) and 5 out of 14 (35.7%).

MGL^−/−^ animals exhibited lung neoplasia (1 high-grade glandular dysplasia and 4 adenocarcinoma). By contrast, only 1 out of 17 (5.9%) wild-type (MGL^+/+^) animal was found to have lung lesion (adenocarcinoma) (Fig. 2c). Thus, the higher proportion of the older MGL-deficient animals exhibited lung neoplasia. Of all adenocarcinomas seen in MGL-deficient animals (5 from MGL^+/−^ and 4 from MGL^−/−^ animals), 4 were papillary predominant adenocarcinomas and 5 were solid predominant adenocarcinomas. Thus, our studies, using animals as a model, demonstrate for the first time that MGL deficiency is an important contributing factor in the development of lung adenocarcinomas.

**Fig. 3:**
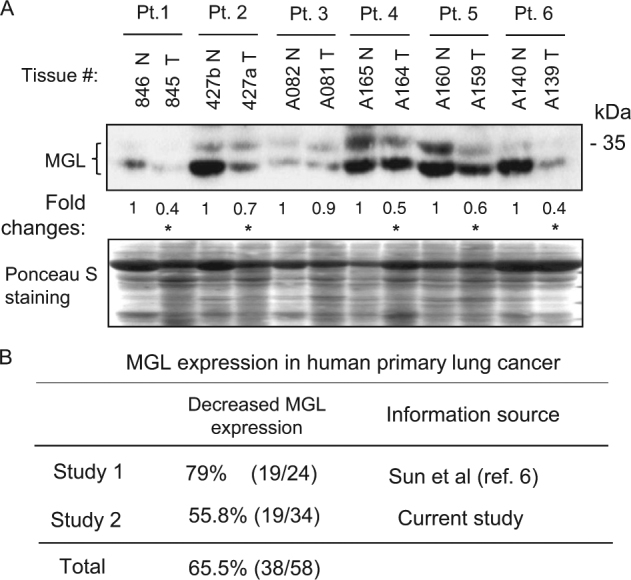
MGL protein expression in patient samples representing matched normal and tumor tissues from the lungs. **a** Western blot analysis showing MGL levels in matched normal (N) and tumor (T) tissues. Pt patient. Ponceau S staining serves as a loading control. Densitometric analyses were performed as described in the Materials and methods. The values for fold reduction in MGL levels in each tumor tissue compared to corresponding matched normal tissue was determined by assigning a value of 1 to normal tissue in each pair of patient samples. These values are written below the western blot image. Tumor tissues showing reduced MGL expression compared to matching normal tissues are indicated by asterisk '*'. **b** Summary of our two MGL expression studies using patient samples representing the matched normal and tumor tissues from the lungs. The results of study #1 were previously reported^6^ and included here to emphasize that MGL is deregulated in human lung cancer. The results of study #2 are generated in the current investigation and thus represent a different cohort of patients. Information on tumor histology for study #1 and study #2 is presented in the Supplementary Tables 1 and 2

We also investigated MGL status in tissues from lung cancer patients including their matching normal tissues. Figure 3a shows the representative results of MGL protein expression analyzed by western blotting and, as is shown, MGL expression was reduced (marked with *) in the majority of lung tumors compared to their matched normal lung tissues. Figure 3b summarizes the results of our two MGL expression studies performed on primary human tumors. The combined analysis of these studies indicates that MGL expression is reduced in 65.5% (38 out of 58) of the primary human lung cancer cases. Tumor histology for both studies is shown in Supplementary Tables 1 and 2. We also analyzed the Firehose/RSEM^7^ and Oncomine^8^ databases that curate data from a number of complementary DNA (cDNA) microarray studies, and our analyses revealed that indeed MGL mRNA reduction occurred in the majority of multiple cancers including lung cancers. Thus, findings from these databases confirm our results.

### MGL-deficient MEFs show oncogenic transformation and increased cell proliferation

**Fig. 4:**
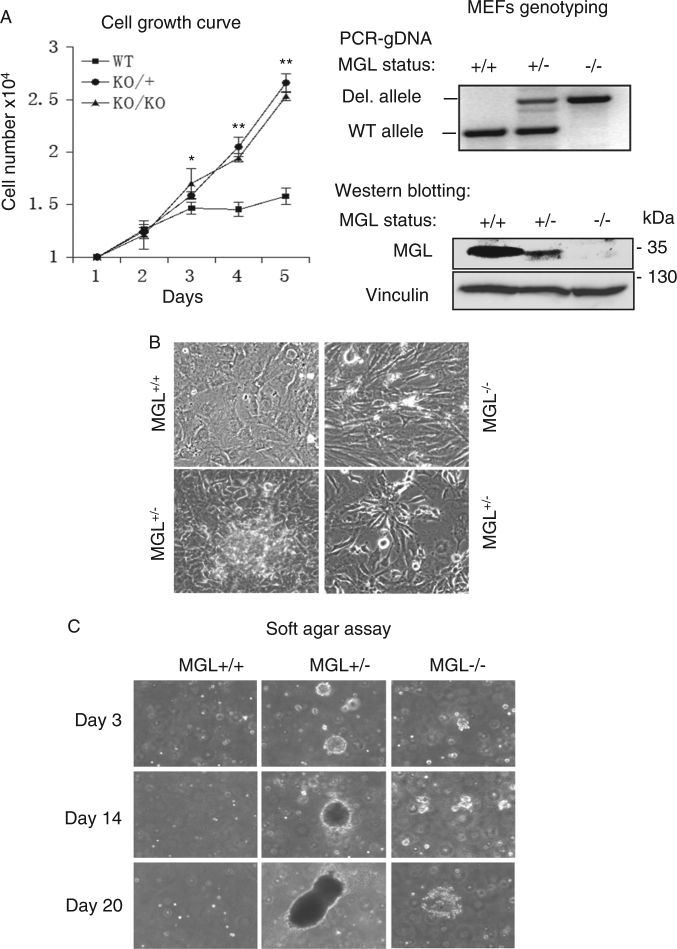
MGL-deficient MEFs display features of oncogenic transformation. **a** Left panel: MGL-deficient MEFs show increased cell proliferation and shortened cell doubling times. Equal numbers of MGL^+/+^, MGL^+/−^ and MGL^−/−^ MEFs (passage 15) (isolated from mice embryos of ~day 14 as described in Materials and methods) were cultured and counted at the indicated times. The presented data are based on experimental results collected from two independent experiments performed in triplicate. *P-value <0.1, **P-value <0.001. Right panel: MGL status of MEFs was confirmed by genomic DNA-PCR (upper panel) and western blot analyses (lower panel). **b**,** c** MGL-deficient (MGL^+/−^ and MGL^−/−^) MEFs but not MGL-proficient (MGL^+/+^) MEFs form foci on cell culture plates (**b**) and exhibit anchorage-independent growth on soft agar (**c**)

Figure 4a shows that MGL-deficient mouse embryonic fibroblasts (MEFs) exhibited significantly increased cell proliferation compared to MGL wild-type MEFs. Cell doubling time in MGL-deficient cells was significantly shortened compared to that in MGL-proficient cells (Fig. 4a). In addition, MGL^+/−^ and MGL^−/−^ MEFs, but not the MGL^+/+^ MEFs, exhibited phenotypic changes of oncogenic transformation. For example, the MGL-deficient MEFs exhibited spindle-like arrangements and formed large numbers of foci under regular culture conditions (Fig. 4b) and also on soft agar (anchorage-independent growth) (Fig. 4c). These results indicate that absence or diminished levels of MGL promote cellular transformation in MGL-deficient MEFs, and the presence of MGL inhibits such oncogenic transformation.

### MGL deficiency activates EGFR-ERK pathway in mouse MEFs and lung tissues

**Fig. 5:**
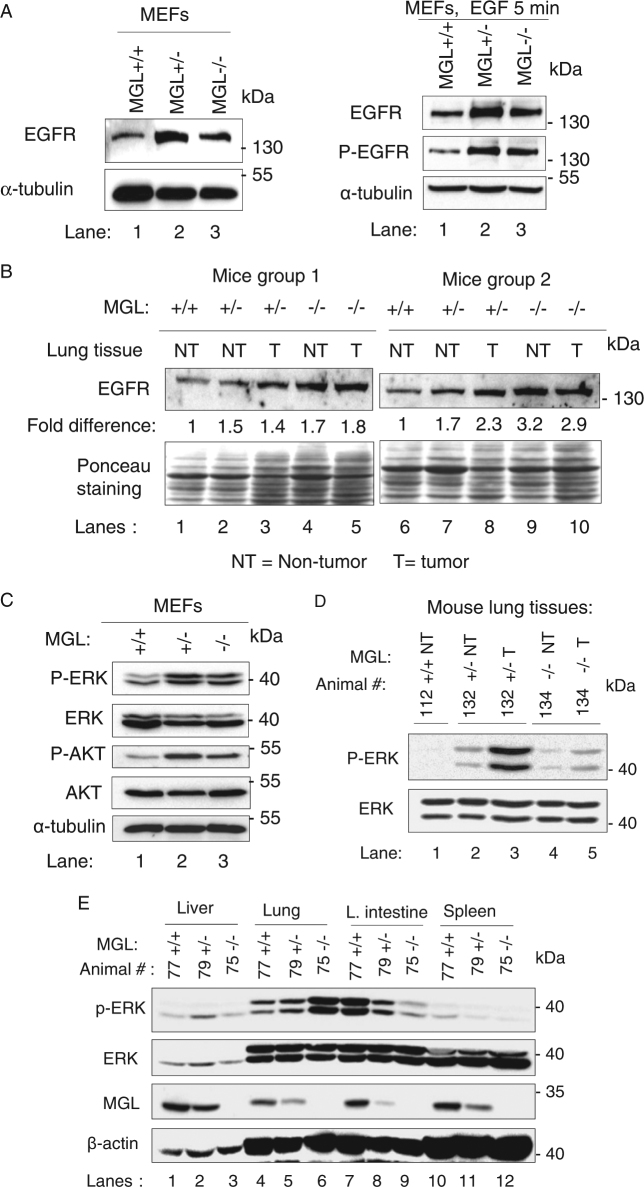
MGL negatively regulates EGFR-ERK pathway in mouse MEFs and lung tissues. **a** EGFR expression is increased in MGL-deficient MEFs without EGF treatment (left). The levels of total EGFR and phospho-EGFR (P-EGFR) are similarly increased in MGL-deficient MEFs with EGF treatment (right, lanes 2 and 3 are compared with lane 1). **b** EGFR levels are increased in the MGL-deficient mouse lung tissues. Tissues from two different groups of mice were used. Western blot analyses were performed as described in Materials and methods. Ponceau S staining of the membranes (lower portions) shows loading of each sample. NT non-tumorous lung tissues. T tumorous lung tissue. Densitometric analyses were performed as described in the Materials and methods. The relative EGFR levels in the lung tissues of MGL^+/+^, MGL^+/−^ and MGL^−/−^ animals in each group of mice are written below the western blot image. **c** Phosphorylation of ERK (P-ERK) and Akt (P-Akt) is increased in MGL-deficient MEFs (lanes 2 and 3) compared to that in MGL-proficient MEFs (lane 1). **d** ERK phosphorylation (P-ERK) levels are increased in the lung tissues of MGL-deficient mice (lanes 2–5) as compared to those in MGL-proficient mouse (lane 1). Numbers on the top indicate each individual animal. NT non-tumor, T tumor. **e** ERK phosphorylation (P-ERK) is increased in the lung tissues, but not in other tissues, of MGL-deficient mice

We also investigated EGFR expression status in MGL-deficient cells and tissues. The EGFR expression and phosphorylation were elevated in MGL^+/−^ and MGL^−/−^ MEFs (Fig. 5a). Furthermore, EGFR levels were also significantly higher in the lung tissues (both tumor (T) and non-tumor (NT)) of MGL-deficient mice (Fig. 5b, lanes 2–5 and 7–10) compared to those in the MGL wild-type animals (lanes 1 and 6). Phosphorylation of ERK and Akt was also increased in the MGL-deficient (+/− and −/−) MEFs, while the levels of total ERK and Akt proteins were not similarly changed (Fig. 5c). Similarly, ERK phosphorylation was increased in lung tissues (T and NT) of MGL-deficient mice (Fig. 5d, lanes 2–5 compared with lane 1). Figure 5e shows ERK status from tissue lysates extracted from an additional group of animals; as is shown, compared to that of the wild-type littermate (lane 4), ERK phosphorylation was modestly increased in the lung tissues of the MGL^+/−^ animal (top panel, lane 5) and strongly increased in the lungs of the MGL^−/−^ animals (lane 6). Surprisingly, MGL deficiency did not lead to increased phospho-ERK levels in several other tissues in MGL-deficient animals (Fig. 5e and Supplementary Figure 2). These results suggest that MGL deficiency in mice affects ERK activity in a tissue-specific manner. In the case of lung tissues, ERK activation due to MGL deficiency likely promotes cell proliferation in animals.

**Fig. 6:**
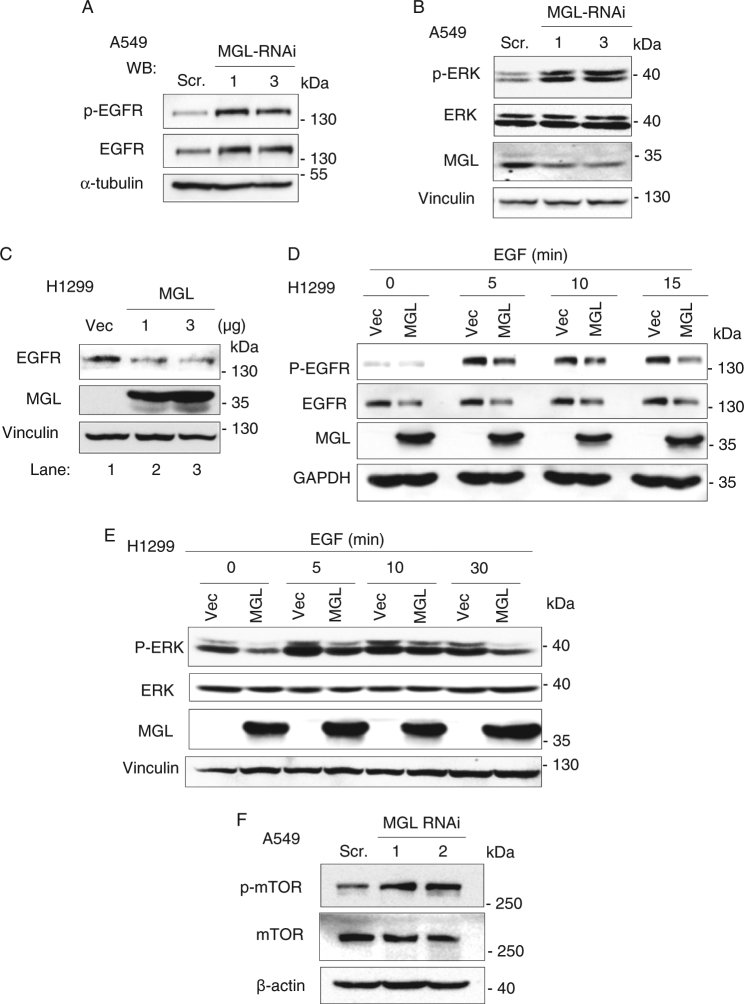
MGL negatively regulates EGFR-ERK and Akt pathway in human lung cancer cells. **a** MGL knockdown increases the levels of total EGFR and phosphorylated-EGFR in A549 lung cancer cells. Two different MGL shRNAs targeting different regions of MGL mRNA were used. Cells were treated with EGF for 5 min before harvesting for western blot analysis. **b** MGL knockdown increases the levels of phosphorylated-ERK (p-ERK) in A549 cells. **c** Overexpression of MGL reduces EGFR levels in H1299 human lung cancer cells. Lane 1: vector alone; lanes 2 and 3: cells transfected with 1 and 3 µg of MGL expression vector, respectively. **d** Overexpression of MGL in H1299 cells negatively regulates the EGFR phosphorylation (p-EGFR) and EGFR protein levels. Vec vector transfected, MGL MGL transfected. Cells were treated with or without EGF (10 ng/ml) for the indicated times. **e** ERK phosphorylation is also inhibited in the H1299 cells overexpressing MGL. Cells were treated with or without EGF (10 ng/ml) for the indicated times. **f** MGL knockdown leads to increased mTOR phosphorylation (p-mTOR) in A549 human lung cancer cells. Two different MGL shRNAs targeting different regions of MGL mRNA were used

Using human NSCLC cell lines, we further investigated MGL-mediated regulation of EGFR and ERK. MGL knockdown (KD) in A549 cells (cells with detectable MGL) increased EGFR protein and phosphorylation levels (Fig. 6a), and also ERK phosphorylation level (Fig. 6b). Conversely, overexpression of MGL in H1299 cells (with no detectable MGL) reduced EGFR protein and phosphorylation levels (Fig. 6c, d), and also levels of ERK phosphorylation (Fig. 6e). MGL KD in human colon (HT29) and breast (MDA-MB-231) cancer cells also led to enhanced ERK phosphorylation (Supplementary Fig. 3). Interestingly, MGL KD also significantly increased phosphorylated mammalian target of rapamycin (mTOR; Akt substrate) levels in A549 cells (Fig. 6f). Together, these results highlight the important role of MGL in regulation of EGFR/ERK and Akt signaling in lung cancer.

### MGL deficiency increases the levels of inflammatory molecules COX-2 and TNF-α

**Fig. 7:**
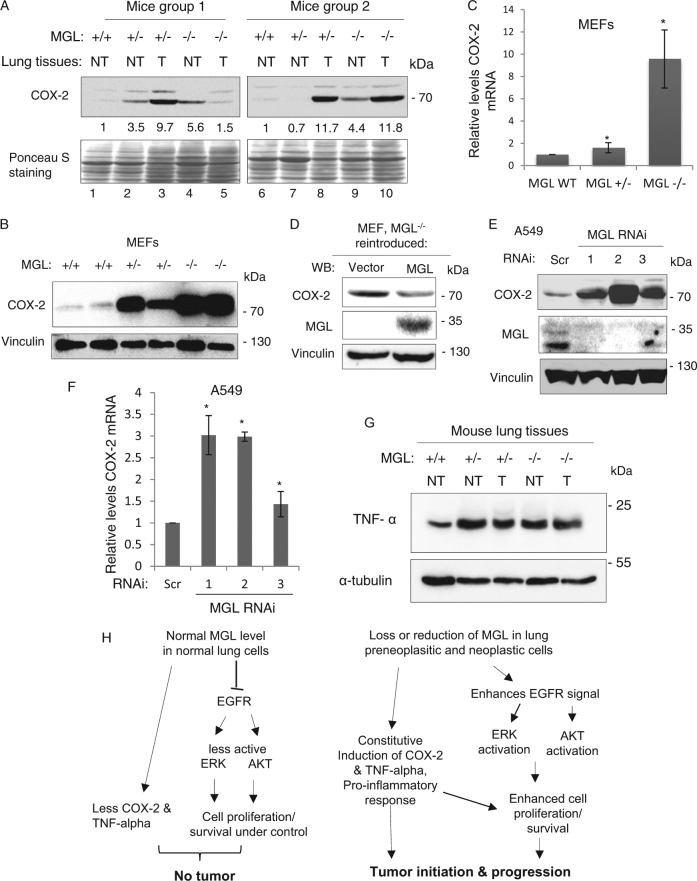
MGL deficiency leads to increased COX-2 levels in MEFs and mouse lung tissues as well as human lung cancer cells. **a** Increased COX-2 levels in the lung tissues of MGL-deficient mice. As indicated, lung tissues of two groups of mice were analyzed. Lanes 1 and 6: tissues from MGL-proficient mice, lanes 2–5 and 7–10: tissues from MGL-deficient mice. Densitometric analyses were performed as described in the Materials and methods. The relative COX-2 levels in lung tissues of MGL^+/+^, MGL^+/−^ and MGL^−/−^ animals in each group of mice are written below the western blot image. Figure 7a and Fig. 5b share the same Ponceau S staining images because the same membranes were separately used for analyzing EGFR (Fig. 5b) and COX-2 (Fig. 7a). Ponceau S staining was done on the membranes immediately after protein transfer and images were taken; the membranes were then cut into two parts based on the molecular markers and separately probed with anti-EGFR and anti-COX-2 antibodies. **b** COX-2 induction in MGL-deficient MEFs. Each sample represents MEFs isolated from different mice embryos. Vinculin serves as a loading control. **c** Increased COX-2 mRNA levels in MGL-deficient MEFs demonstrated by qRT-PCR. mRNAs extracted from MGL-proficient and -deficient MEFs were reverse transcribed and subjected to quantitative PCR (qRT-PCR). The qRT-PCR data represent results collected from three-independent experiments performed in triplicate. *P-value <0.05. **d** Re-expression of MGL (pSRα-MGL expression vector) in MGL knockout (MGL^−/−^) MEFs suppresses COX-2 levels. **e**, **f** MGL knockdown (by three different MGL shRNAs, 1, 2 and 3) in A549 human lung cancer cells increases COX-2 protein (**e**) and mRNA (**f**) levels. The qRT-PCR data represent results collected from three-independent experiments performed in triplicate. *P-value <0.05. **g** Increased TNF-α levels in the lung tissues of MGL-deficient mice. NT non-tumorous lung tissues, T tumorous lung tissue. **h** Schematic illustration of the proposed model depicting MGL involvement in the regulation of EGFR/ERK and COX-2/TNF-α in normal and cancer cells as discussed in the text of Discussion section

Figure 7a shows that cyclooxygenase-2 (COX-2) expression was also elevated in 3 out of 4 NT and 4 out of 4 T lung tissues from MGL^+/−^ and MGL^−/−^ mice (lanes 2–5 and 8–10) compared to those of MGL wild-type (MGL^+/+^) mice (lanes 1 and 6). Strong COX-2 induction was also noted in MGL-deficient MEFs (Fig. 7b). Real-time reverse transcription PCR (RT-PCR) analyses revealed that COX-2 mRNA was also elevated in the MGL-deficient MEFs, especially in the MGL^−/−^ MEFs (Fig. 7c). Conversely, re-introduction of MGL into the MGL-deficient MEFs (MGL^−/−^) resulted in reduced COX-2 levels (Fig. 7d). In A549 cells, MGL knockdown also led to increased COX-2 protein and mRNA levels (Fig. 7e, f). Levels of another pro-inflammatory molecule tumor necrosis factor-α (TNF-α) were also elevated in MGL-deficient lung tissues (Fig. 7g). Together, these results indicate that the activation of pro-inflammatory state in the lung tissues may be important for lung tumorigenesis in MGL-deficient animals.

## Discussion

In this study, we demonstrate that MGL deficiency in mice leads to tumor development in several organs including lungs, lymphoid tissues, liver and soft tissues. Although tumors were noted in various organs, lungs were the most frequent site of neoplastic changes. In the older animals (16–24 months old), neoplastic changes in lungs were noted in 29.2% of MGL^+/−^ and 35.7% of MGL^−/−^ mice compared to only 5.9% in MGL^+/+^ animals (Fig. 2c). These novel findings indicate that MGL plays an important role in lung tumorigenesis in animals. Currently, the mechanisms underlying lung tumorigenesis are not fully understood, especially lung cancer development in non-smokers. Our results show that MGL-deficient (MGL^+/−^ and MGL^−/−^) animals, without any exposure to carcinogenic/inflammatory substances, developed premalignant and malignant changes (adenocarcinoma) in their lung tissues. Abnormality in lung tissues due to MGL deficiency noted in animals is also recapitulated in humans. For example, our results demonstrate that insufficient MGL expression (either absent or decreased) is common (~65%) in human primary lung cancers compared to matched normal lung tissues (Fig. 3b). Searches in the Oncomine Cancer Microarray database (https://www.oncomine.org)^8^ reveal that in several studies^9–11^ of large-scale microarray analyses, MGL expression is found to be reduced in the majority of lung cancers compared to normal tissues. MGL reduction is not restricted to one cell type but involved in many types of human lung cancers, i.e., squamous cell lung cancer, adenocarcinoma, small-cell and large-cell lung cancers (see ref. ^8^, and Supplementary Tables 1 and 2). Currently, it is not clear why the MGL-deficient animals develop adenocarcinomas but not the other types such as squamous cell carcinomas. It is possible that for squamous cell cancer or other types of lung cancer to develop in MGL-deficient animals, additional genetic changes and/or smoking-like environment may be required.

We also identified important molecular changes associated with MGL deficiency including increased expression and phosphorylation of EGFR (Figs. 5 and 6), and induction of COX-2 and TNF-α (Fig. 7). EGFR activation due to EGFR mutation is considered as one of the important oncogenic events in lung tumorigenesis. EGFR is reported to be overexpressed in 22–81% of NSCLC^12–14^; thus, EGFR overexpression has also been implicated in NSCLC pathogenesis^5^. Accordingly, EGFR is now an important target for lung cancer therapy^2,4^. Our present study has identified MGL to be a novel negative regulator of EGFR. For example, MGL depletion led to induction of EGFR in MGL-deficient MEFs and lung tissues as well as in MGL KD lung cancer cells (Figs. 5 and 6). Although the exact mechanisms via which MGL suppresses EGFR remain unclear, our preliminary results indicate that EGFR mRNA levels are elevated in MGL-deficient MEFs (data not shown). Thus, MGL appears to modulate EGFR expression, at least in part, at the mRNA levels. We also show that MGL negatively regulates both ERK and Akt signals (Figs. 5 and 6); both of these pathways are known to positively modulate EGFR expression. For example, EGFR promoter is known to be regulated by oncoproteins, such as AP1 (c-Jun/Fos) and β-catenin^15–17^ that are downstream targets of the ERK and Akt pathways^18,19^. It is therefore possible that MGL may modulate EGFR expression via its negative regulation of ERK and Akt pathways. It is also possible that MGL may modulate EGFR via a transcription-independent mechanism, since EGFR levels are also modulated by protein–protein interaction, endocytosis and protein turnover/degradation (reviewed in ref. ^20^). Further in-depth studies are needed to elucidate the exact mechanisms via which MGL regulates EGFR.

We also found that elevated COX-2 expression is one of the important molecular changes identified in MGL-deficient cells. COX-2 protein expression was significantly elevated in the lung tissues and the MEFs of the MGL-deficient animals as well as in the MGL KD human lung cancer cells (Fig. 7). COX-2 mRNA levels were also significantly increased in MGL-deficient MEFs and MGL KD lung cancer cells. These findings suggest that MGL regulation of COX-2 occurs, at least in part, at the mRNA level. However, we also note that the extent of changes in COX-2 mRNA and protein levels are not always congruent, thus raising the possibility of both transcriptional and post-transcriptional mechanisms. Future studies are expected to delineate these possibilities.

COX-2 is known to be a critical modulator of inflammatory response and its expression is commonly upregulated during inflammation; constitutive overexpression of COX-2 has also been implicated in a variety of human malignancies, including lung cancer^21,22^. Studies have shown that the use of COX-2 inhibitors for more than 1 year significantly reduced lung cancer risks^23^. Thus, elevation of COX-2 levels appears to be one of the important events in lung cancer development. Likewise, induction of COX-2 due to MGL insufficiency, as noted in our study, could also be one of the important events leading to lung cancer development in the MGL-deficient animals. In agreement with this notion, we found that the expression of pro-inflammatory cytokine TNF-α was also significantly elevated in MGL-deficient lung tissues (Fig. 7g). Inflammation is one of the common features underlying the pathogenesis of cigarette smoke-associated disease, including lung cancer. Conceivably, the prolonged and consistent induction of pro-inflammatory molecules such as COX-2 and TNF-α could lead to chronic inflammation and damage in the lung tissues and such condition, mimicking smoking, could favor tumor development in MGL-deficient lung tissues.

Based on our current findings, we propose a model depicting the MGL involvement in tumorigenesis (Fig. 7h). As is shown, in normal lung cells, MGL levels are in abundance; accordingly, MGL tends to reduce EGFR expression, suppress ERK and Akt/mTOR phosphorylation and keep cell proliferation/survival under control. In addition, MGL suppresses COX-2 and TNF-α and inhibits inflammatory responses. In MGL-deficient cells (i.e., in lung and other tissues), MGL deficiency would lead to higher activities of EGFR, ERK and Akt/mTOR, as well as induction of pro-inflammatory molecules COX-2 and TNF-α. Prolonged and cumulative effects of these overly active signaling molecules would eventually lead to tumor initiation/progression.

There are different views about MGL role in tumorigenesis; for example, a report by Nomura et al.^24^ suggested MGL to be an oncogenic protein involved in tumor metastasis due to its lipase activity. Interestingly, according to the Firehose/RSEM and Oncomine public databases, MGL expression is significantly lower in the lung tumor tissues, but higher in some kidney cancers^7,8^. Therefore, it is possible that MGL may have dual roles as a tumor suppressor or oncoprotein, depending on the tissue types. Several lines of published evidence indicate that certain proteins, such as Notch, Spleen Tyrosine Kinase, Sirtuins, WT-1 and transforming growth factor-β, exhibit both tumor suppressive and oncogenic functions that depend on cell/tissue types or developmental stages of tumors^25–29^. In our MGL knockout (MGL^−/−^) animals, MGL lipase activity is expected to be completely abolished due to the gene deletion; however, 60% (21 out of 35) of MGL^−/−^ mice developed tumors in various tissues (Table 1). Interestingly, a recent study by Rajasekaran et al.^30^ showed that MGL expression was undetectable in primary human hepatocellular carcinoma (HCC) samples (Oncomine database also shows similar findings) and overexpression of MGL inhibited HCC cell growth in vitro and in xenografted tumors^30^. These findings are consistent with the tumor suppressor role for MGL, and in line with our previous study^6^ and current findings indicating that MGL-deficient mice also developed liver tumors.

In summary, using the MGL-deficient animal model, we report for the first time that MGL functions as a tumor suppressor particularly in context to lung cancer. Based on our results, we propose that absent or reduced MGL expression is one of the important molecular alterations underlying human lung cancer development. Our results also suggest that the MGL-deficient animals are a unique mouse model that can be used, in the future, to develop newer targeted therapies for human malignancies particularly lung adenocarcinomas.

## Materials and methods

### Animal studies

MGL knockout mice were generated commercially by the Gene Targeting and Transgenic Facility (GTTF) at the University of Connecticut Health Center (Farmington, CT). The details of MGL gene targeting strategy are outlined in Supplementary Figure 1. Studies involving animals were performed in accordance with the guidelines of the Institutional Animal Care and Use Committee of the State University of New York (SUNY) Upstate Medical University. Male and female C57BL/6 mice of mixed background were used in these studies. For histological studies, the tissues were fixed in 4% paraformaldehyde and hematoxylin and eosin (H&E) stained as previously described^6^ and reviewed by two or three pathologists (SL, CC and RR)^31^.

For genotyping of mice and MEFs, genomic DNAs extracted from tails of mice (<20 days) or MEFs were subjected to PCR reactions using the following primers: Lox gtF 5′-AGCTGAGGTCCATGCCTTAG-3′; FrtgtR 5′-CTAAGAGTGTATTACAGCAC-3′; FrtgtF: 5’-AATCTCAGATGAACCAGCAC-3′. The PCR products of 354 and 540 bp fragments indicated the presence of the MGL wild-type and MGL knockout alleles, respectively. For tissue studies, animal organs were collected from the euthanized  animals and protein analyses were performed as previously described^6^.

### Antibodies, expression vectors and reagents

The phospho-ERK1/2 (Thr202/Tyr204), ERK1/2, phospho-AKT (Ser473), AKT, phospho-EGFR (Tyr1068) phosphor-mTOR (Ser2248) antibodies were from Cell Signaling (Danvers, MA); EGFR antibody was from Abcam (Cambridge, MA). MGL antibody was generated in our laboratory as previously described^6^. The β-actin and α-tubulin antibodies were from Sigma (St. Louis, MO). GAPDH, vinculin, TNF-α and COX-2 antibodies were from Santa Cruz Biotechnologies (Dallas, TX). pDsRedN1-MGL vector (MGL-RFP) has been previously described^6^. For pSRα-HA-S-tagged MGL expression vector, complete human MGL ORF was subcloned into mammalian pSRα-HA-S vector (contains HA and S tags) and DNA sequencing was performed to confirm the integrity of MGL sequence.

### Cell culture and cell doubling time assay

MEFs were prepared from mice embryos between days 12.5 and 14.5 of gestation according to the procedures described^32^. MEFs were maintained in Dulbecco’s modified Eagle’s medium (DMEM) (Cellgro/Mediatech, Herndon, VA) supplemented with 10% fetal bovine serum (FBS) (Gemeni Bioproducts Inc., Calabasas, CA). Human cancer cell lines A549 (NSCLC), H1299 (NSCLC) and HeLa (cervical cancer) were obtained from the National Institutes of Health (NIH) and regularly maintained in DMEM supplemented with 10% FBS. To perform cell doubling time assay, MEFs of all MGL genotypes were seeded at equal numbers, harvested at the indicated times (up to 5 days) and cell numbers were then counted.

### Soft agar assay

The assays were performed in 6-well plates using the low-melt agarose (Bio-Rad, Hercules, CA). The bottom layer was 0.8% agar diluted in DMEM with 20% FBS. For the top layer, cells were first suspended in 0.48% agar in DMEM with 20% FBS and then plated on top of the bottom layer. Additional 1 ml of DMEM was added to each well. Cells were incubated in humidified 5% CO_2_ incubator at 37 °C for 2–3 weeks.

### Lentivirus-mediated shRNA silencing

MGL knockdown was achieved by the lentivirus-mediated small hairpin RNA (shRNA) expression as we previously described^6^. Three different nucleotide sequences targeting the human MGL were as follows: shRNA1 5′-ccaggacaagactctcaagat-3′; shRNA2 5′-caactccgtcttccatgaaat-3′; and shRNA3 5′-ccaatcctgaatctgcaacaa-3′. The scramble shRNA construct was commercially obtained from Addgene, Inc. (Cambridge, MA). Lentivirus preparation, expansion and infection were performed per protocol provided by Addgene.

### Western blot

Western blot (WB) analyses were performed as previously described^6^. Densitometric analyses were performed for all WB membranes stained with S-Ponceau using total amount of stained protein in each lane. The levels of a specific protein (i.e., EGFR, COX-2 and MGL) are normalized with respect to corresponding S-Ponceau levels. The fold differences are given under the relevant WB images.

### Quantitative RT-PCR for analyzing mRNA expression

Quantitative real-time PCR (qRT-PCR) assays were performed as previously described^33^ using the iScript cDNA synthesis kit and iQ SYBR Green super-mix from BIO-RAD (Hercules, CA). C(T) values for COX-2 were normalized to the C(T) values of GAPDH mRNA in the same sample from either human or mouse cells. The detailed information about primers used for qRT-PCR studies is listed in Supplementary Fig. 4.

### Statistical analysis

Results are expressed as mean±s.e. Student’s *t*-test and one-way analysis of variance were used for statistical analysis in continuous data. Logistic regression and *z*-test were used for statistical analysis in categorical data. Statistical powers were calculated by general linear model in SPSS.

## Electronic supplementary material

Supplementary Figs and Tables
