# Supplementary material for: Monoglyceride lipase gene knockout in mice leads to increased incidence of lung adenocarcinoma
Source: Cell Death Dis. 2018 Jan 18;9(2):36. doi: 10.1038/s41419-017-0188-z (PMC5833374; doi:10.1038/s41419-017-0188-z)
Supplement: Supplementary file 1 — Supplementary Figs and Tables [file 41419_2017_188_MOESM1_ESM.pdf]

Supplementary Figure 1

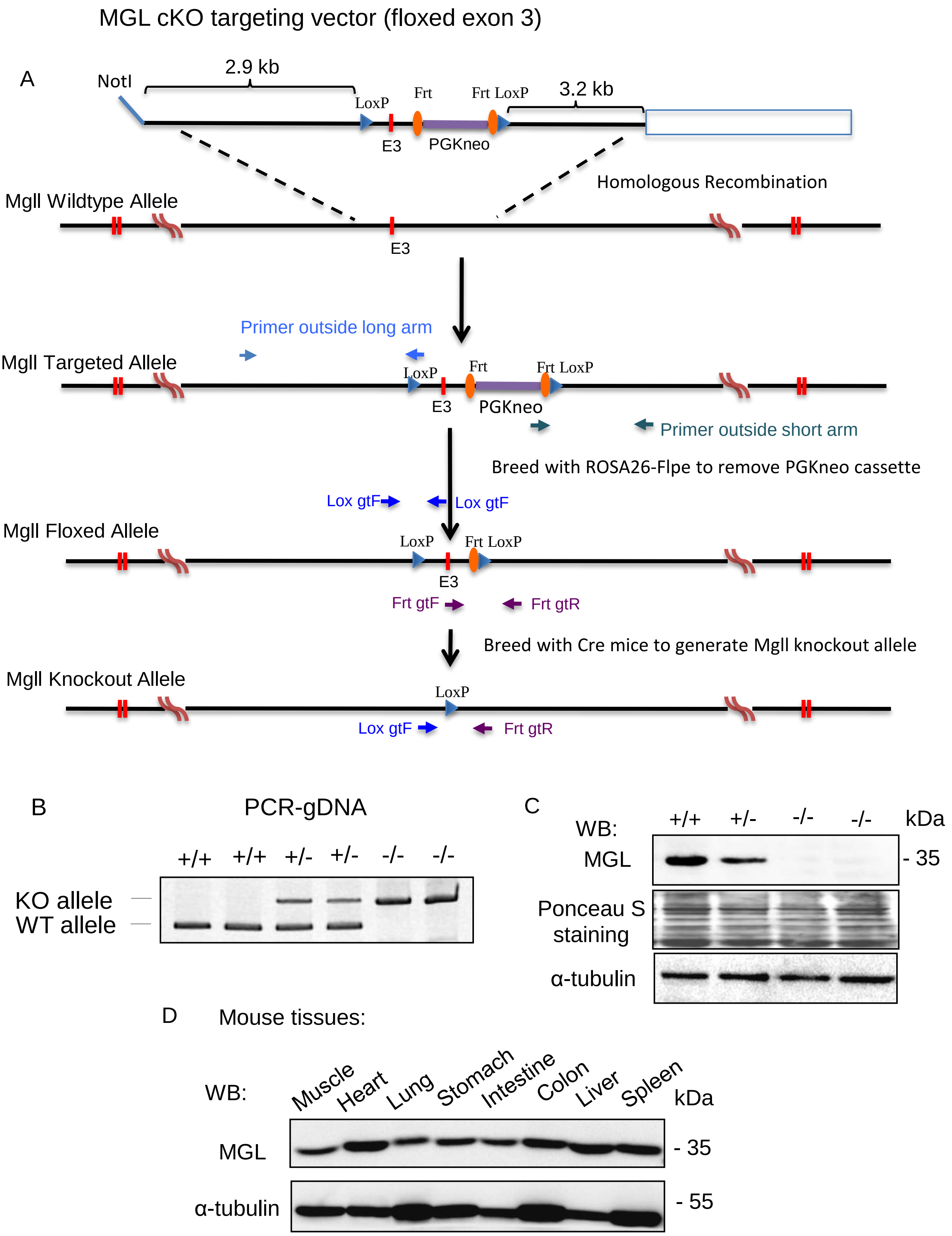

Supplementary Figure 1
(legend)

Supplementary Figure 1. Schematic diagram of MGL knockout (cKO) targeting vector and strategy. A. The targeting vector was designed as shown at the top with LoxP sites flanking the exon 3 of MGL. The selection marker PGKneo was also inserted into the flanked region. The vector was used to transfect mouse embryonic stem (ES) cells. The subsequent cellular homologous recombination resulted in ES clones with MGL allele containing the LoxP sites and selection marker. Breeding of mouse containing the modified MGL allele with ROSA26-Flpe mouse then removed the PGKneo cassette. Following further breeding with Cre mice, MGL knockout allele (MGL+/-) was generated. MGL-/- mice were generated by crossing MGL+/- mice. Inbreeding among the MGL hemizygous mice generated mice with three different genotypes including the wild-type (MGL+/+), hemizygous (MGL+/-) and homozygous (MGL-/-)-deficient mice. B. Representative genotyping results of genomic DNA-PCR of animal tail tissues. PCR primers used in the reactions are described in the Materials and Methods. C. MGL protein expression in mice tissues as detected by western blot analysis using MGL-specific antibody. Ponceau S staining and α-tubulin show protein loading of the membrane. D. Western blot analysis showing MGL protein expression in the indicated tissues of a wild-type (MGL+/+) mouse.

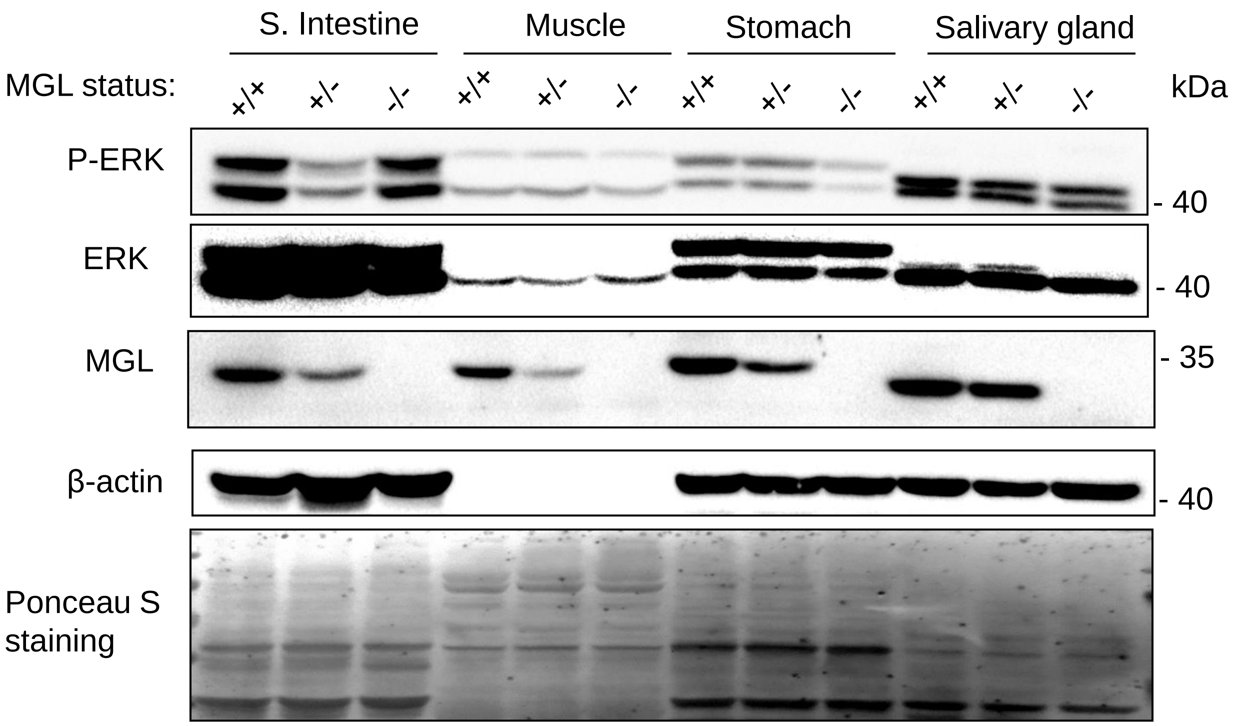

Supplementary Figure 2

WB analysis of ERK, P-ERK in multiple mouse tissues. MGL status of each type of tissues are indicated. Both β-actin and Ponceau S staining serve as the loading controls.

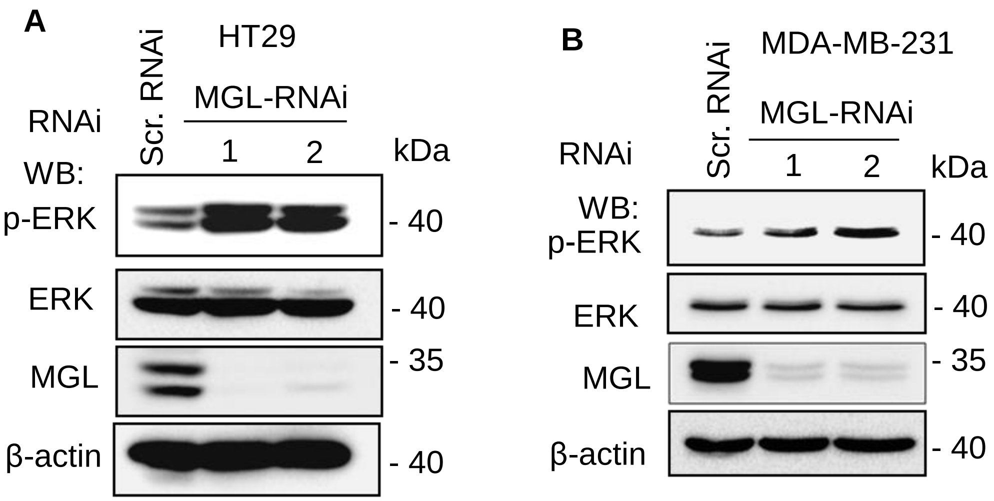

Supplementary Fig. 3 MGL knockdown enhanced ERK phosphorylation in HT29 colon cancer cells (A) and MBA-MD-231 breast cancer cells (B). Two different MGL shRNAs (#1 and #2) were used for MGL knockdown.

Primers used for qRT-PCR analyses:

Gene	Primers	Sequences
EGFR (human)	forward primer:	5’-CGTGGCAAGTCCCCCAGTGA-3’
	reverse primer:	5’-GCAGACCAGGCAGTCGCTCTC-3’
EGFR (mouse)	forward primer:	5’-AGGCACAAGTAACAGGCTCAC-3’
	reverse primer:	5’-AAGGTCGTAATTCCTTT GCAC-3’
COX-2 (human)	forward primer:	5’-CCTTCCTCCTGTGCCTGATG-3’
	reverse primer:	5’-ACAATCTCATTTGAATCAGGAAGCT-3’
COX-2 (mouse)	forward primer:	5’-CAAGGGAGTCTGGAACATTG-3’
	reverse primer:	5’-ACCCAGGTCCTCGCTTATGA-3’
GAPDH (human)	forward primer:	5’-GAGTCAACGGATTTGGTCGT-3’
	reverse primer:	5’-TTGATTTTGGAGGGATCT CG-3’
GAPDH (mouse)	forward primer:	5’-CGACTTCAACAGCAACTCCCACTCTTCC-3’
	reverse primer:	5’-TGGGTGGTCCAGGGTTTCTTACTCCTT-3’

Supplementary Table 1. Clinicopathological features of primary lung cancer samples exhibiting altered expression of MGL at the mRNA levels.

Patient No.	Age (yrs)	Sex	Tumor Type	MGL Status
1	66	Male	Squamous cell carcinoma	Decreased
2	69	Female	Bronchiolo-alveolar adenocarcinoma	Increased
3	73	Male	Squamous cell carcinoma	Decreased
4	65	Male	Squamous cell carcinoma	Increased
5	64	Male	Squamous cell carcinoma	Decreased
6	52	Male	Malignant carcinoid tumor	Decreased
7	66	Male	Squamous cell carcinoma	Decreased
8	57	Male	Squamous cell carcinoma	Decreased
9	46	Male	Adenocarcinoma	Decreased
10	65	Male	Adenocarcinoma	Decreased
11	46	Male	Squamous cell carcinoma	Increased
12	61	Male	Bronchiolo-alveolar adenocarcinoma	Decreased
13	65	Female	Squamous cell carcinoma	Decreased
14	62	Male	Adenocarcinoma	Increased
15	59	Female	Adenocarcinoma	Decreased
16	44	Female	Bronchiolo-alveolar adenocarcinoma	Decreased
17	75	Male	Squamous cell carcinoma	Decreased
18	33	Female	Malignant carcinoid tumor	Decreased
19	62	Male	Adenocarcinoma	Decreased
20	60	Male	Squamous cell carcinoma	Decreased
21	58	Male	Squamous cell carcinoma	Decreased

This Table contains information reported in Study # 1 (ref. 6); the histological information on these samples was not reported in ref. 6; included here for overall evaluation of MGL expression in primary lung cancer.

Supplementary Table 2. Clinicopathological features of primary lung cancer samples exhibiting altered expression of MGL at the protein levels.

Patient No.	Age (yrs)	Sex	Tumor Type	MGL Status
1	72	Female	Squamous cell carcinoma	Decreased
2	57	Female	Adenocarcinoma	Decreased
3	77	Female	Adenocarcinoma	Unchanged
4	64	Male	Squamous cell carcinoma	Decreased
5	77	Male	Adenocarcinoma	Decreased
6	59	Female	Squamous cell carcinoma	Decreased
7	72	Male	Squamous cell carcinoma	Unchanged
8	76	Male	Squamous cell carcinoma	Decreased
9	48	Male	Adenocarcinoma	Increased
10	70	Male	Adenocarcinoma	Increased
11	70	Female	Squamous cell carcinoma	Unchanged
12	68	Male	Adenocarcinoma	Decreased
13	68	Male	Squamous cell carcinoma	Decreased
14	76	Male	Squamous cell carcinoma	Decreased
15	80	Female	Squamous cell carcinoma	Increased
16	66	Male	Squamous cell carcinoma	Decreased
17	60	Male	Adenocarcinoma	Unchanged
18	78	Male	Squamous cell carcinoma	Unchanged
19	74	Female	Adenocarcinoma	Increased
20	68	Male	Adenocarcinoma	Decreased
21	47	Male	Adenocarcinoma	Decreased
22	63	Male	Adenocarcinoma	Decreased
23	60	Male	Adenocarcinoma	Decreased
24	66	Female	Adenocarcinoma	Unchanged
25	73	Female	Squamous cell carcinoma	Decreased
26	53	Male	Adenocarcinoma	Decreased
27	74	Male	Adenocarcinoma	Increased
28	67	Male	Squamous cell carcinoma	Increased
29	75	Female	Adenocarcinoma	Decreased
30	68	Male	Adenocarcinoma	Increased
31	54	Male	Adenocarcinoma	Unchanged
32	76	Male	Adenocarcinoma	Increased
33	57	Female	Adenocarcinoma	Decreased
34	75	Female	Adenocarcinoma	Decreased

Results reported in this Table represent Study # 2 i.e. present manuscript.
